# Sensitivity to Cisplatin in Head and Neck Cancer Cells Is Significantly Affected by Patient-Derived Cancer-Associated Fibroblasts

**DOI:** 10.3390/ijms22041912

**Published:** 2021-02-15

**Authors:** Barbora Peltanova, Marketa Liskova, Jaromir Gumulec, Martina Raudenska, Hana Holcova Polanska, Tomas Vaculovic, David Kalfert, Marek Grega, Jan Plzak, Jan Betka, Michal Masarik

**Affiliations:** 1 Department of Pathological Physiology, Faculty of Medicine, Masaryk University, Kamenice 5, CZ-625 00 Brno, Czech Republic; bpeltanova@seznam.cz (B.P.); liskovam@tiscali.cz (M.L.); j.gumulec@med.muni.cz (J.G.); m.raudenska@gmail.com (M.R.); hana.polanska@gmail.com (H.H.P.); 2 Department of Physiology, Faculty of Medicine, Masaryk University, Kamenice 5, CZ-625 00 Brno, Czech Republic; 3 Department of Chemistry and Biochemistry, Mendel University, Zemedelska 1, CZ-613 00 Brno, Czech Republic; 4 Department of Chemistry, Faculty of Science, Masaryk University, Kotlarska 2, CZ-611 37 Brno, Czech Republic; vaca_777@yahoo.com; 5 Department of Otorhinolaryngology and Head and Neck Surgery, University Hospital Motol, First Faculty of Medicine, Charles University, V Uvalu 84, 150 06 Prague 5, Czech Republic; david.kalfert@fnmotol.cz (D.K.); Jan.Plzak@fnmotol.cz (J.P.); jan.betka@fnmotol.cz (J.B.); 6 Department of Pathology and Molecular Medicine, 2nd Faculty of Medicine, Charles University and University Hospital Motol, V Uvalu 84, 150 06 Prague 5, Czech Republic; marek.grega@fnmotol.cz; 7 BIOCEV, First Faculty of Medicine, Charles University, Prumyslova 595, CZ-252 50 Vestec, Czech Republic

**Keywords:** head and neck cancer, cancer-associated fibroblasts, cisplatin, treatment resistance, cancer recurrence, patient-derived cell cultures, coculture

## Abstract

Cancer-associated fibroblasts (CAFs) are one of the most abundant and critical components of the tumor stroma. CAFs can impact many important steps of cancerogenesis and may also influence treatment resistance. Some of these effects need the direct contact of CAFs and cancer cells, while some involve paracrine signals. In this study, we investigated the ability of head and neck squamous cell carcinomas (HNSCC) patient-derived CAFs to promote or inhibit the colony-forming ability of HNSCC cells. The effect of cisplatin on this promoting or inhibiting influence was also studied. The subsequent analysis focused on changes in the expression of genes associated with cancer progression. We found that cisplatin response in model HNSCC cancer cells was modified by coculture with CAFs, was CAF-specific, and different patient-derived CAFs had a different “sensitizing ratio”. Increased expression of *VEGFA*, *PGE2S*, *COX2*, *EGFR*, and *NANOG* in cancer cells was characteristic for the increase of resistance. On the other hand, *CCL2* expression was associated with sensitizing effect. Significantly higher amounts of cisplatin were found in CAFs derived from patients who subsequently experienced a recurrence. In conclusion, our results showed that CAFs could promote and/or inhibit colony-forming capability and cisplatin resistance in HNSCC cells via paracrine effects and subsequent changes in gene expression of cancer-associated genes in cancer cells.

## 1. Introduction

Head and neck squamous cell carcinomas (HNSCC) are the sixth most common cancer worldwide. HNSCC tumors emerge in the upper aero-digestive tract (including the oral cavity, pharynx, and larynx). Surgery and radiotherapy are effective in the treatment of early-stage tumors (stage I and II). Nevertheless, about 70% of patients are diagnosed with an advanced stage III or IV. The locoregional disease recurs in 60% of patients and metastatic disease arises in 15–25% [1]. For these reasons, chemotherapy (alone or in combination with radiotherapy) has an important role in the treatment of HNSCC. Currently, the key anticancer drug for advanced HNSCC is still cisplatin (cis-diamminedichloroplatinum; CDDP) [2,3]. Nevertheless, HNSCC represents a heterogeneous group of tumors with varying levels of cisplatin resistance. CDDP administration in resistant cases would provide almost no curative effect but could increase the chance of adverse side effects and disease progression. Recently, it was shown that stromal cells in tumor tissue may modulate the resistance to chemotherapy [4]. Moreover, it was shown that drug resistance is likely to be reversible by removal of the tumor-supporting stromal cells [5]. The effect of the tumor microenvironment (TME) on resistance to therapy is easy to understand since different soluble factors produced by the TME can trigger pro-survival pathways in tumor cells, especially when damaged by chemotherapy [6,7]. The most represented cells within the stroma are cancer-associated fibroblasts (CAFs).

In this study, we investigated the ability of HNSCC patient-derived CAFs to promote or inhibit the colony-forming capability of HNSCC cells, and the effect of cisplatin on this promoting or inhibiting influence. Subsequent analysis on FaDu and Detroit562 cell lines focused on changes in the expression of genes associated with: (1) pluripotency (*NANOG*, *SOX2*, *POU5F*, *SNAIL*); (2) cell division (*FOLR1*); (3) proliferative activity of tumor cells (*MKI67*); (4) angiogenesis (*VEGFA*); (5) acquisition of autonomous proliferative signalling (*EGFR*, *EGF*); (6) immune response (*COX2*, *CCL2*, *IL6*, *EP3*, *PGE2S*); and (7) cell cycle, and cell death modifications (*BCL2*, *BIRC5*, *NFKB1*, *CAV1*).

## 2. Results

### 2.1. Clinical Characterization of Patients and Tumors Used for CAFs Preparation

Primary cultures were created from seven head and neck squamous cell primary tumors. Of those, one sample was from a female and three were p16-positive. Tumor location was as follows: three laryngeal, three oropharyngeal, and one hypopharyngeal. Two patients exhibited recurrence after successful therapy. Patient characteristics are shown in Table 1.

### 2.2. Lineage Specificity of Patient-Derived CAFs and Preparation of Conditioned Media

**Figure 1:**
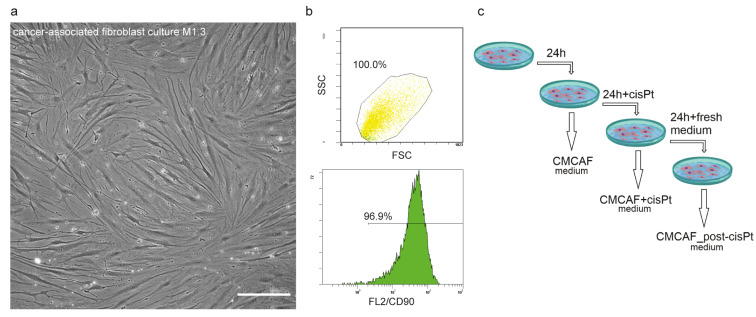
Preparation and characterization of cancer-associated fibroblasts (CAFs) and CAF-conditioned media. (**a**) Representative phase-contrast image of CAF culture, 20× magnification, scalebar indicates 100 µm. (**b**) Verification of fibroblast phenotype by CD90 status. Forward scatter and side scatter population (top) and FL2 histogram. (**c**) Scheme of CAF-derived conditioned media preparation for indirect coculture experiments. After the cells (CAFs) reached optimal confluence, their culture media were replaced with fresh media. After 24 h the conditioned media were collected (CMCAF) and replaced with media containing 5 µM cisplatin. After 24 h the media were collected (CMCAF+cisplatin) and replaced with fresh media, which were collected after an additional 24 h (CMCAF_post-cisplatin).

Patient-derived CAFs were prepared as early passage cell populations created by the overgrowth of the origin nal patient-derived primary culture from stromal fibroblasts presented in this primary culture. Population overgrowth by fibroblasts was successfully reached after two passages. The passages of CAFs used for further analyses ranged from one to four. The CAFs cell populations showed a typical spindle-shaped cell morphology (Figure 1a). Our selection method was confirmed by flow cytometry of three randomly selected early passage CAFs. The CD90 (a CAFs marker [8]) positivity of these selected cultures ranged between 95.2% and 97.2% (Figure 1b, Supplementary Figure S1). This indicated the successful establishment of the CD90+ CAF population.

After the cells (CAFs) reached optimal confluence, their culture media were replaced with fresh media. After 24 h the conditioned media were collected (CMCAF) and replaced with media containing 5 µM cisplatin [9]. After 24 h the media were collected (CMCAF+cisplatin) and replaced with fresh media, which were collected after an additional 24 h (CMCAF_post-cisplatin) (see Figure 1c). These conditioned media were further used for gene expression experiments and platin amount assay. 

### 2.3. Patient-Derived CAFs Affect the Colony-Forming Capability of Cancer Cells

**Figure 2:**
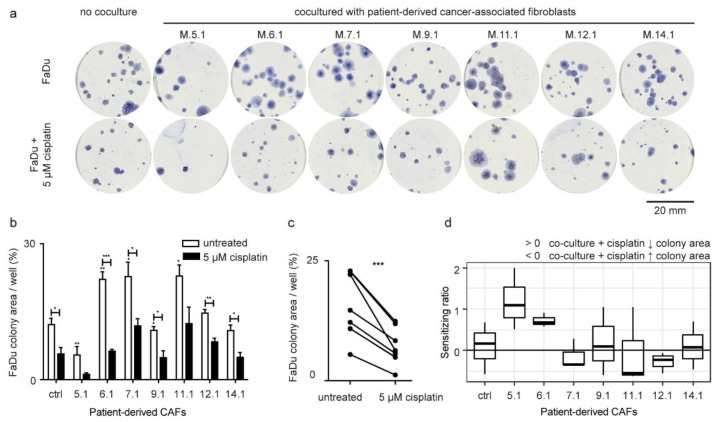
Effect of patient-derived CAFs on the area of cancer cell colonies. (**a**) Representative Coomassie-blue stained FaDu colonies in six-well plates. Patient-derived CAFs either promoted, inhibited, or had no effect on the formation of FaDu colonies, as shown by the different size of their area compared to control. (**b**) The total area of colonies relative to well area (percentage of area covered), not cocultured (ctrl) FaDu cells, and FaDu cocultured with CAFs (5.1–14.1). The asterisk above white bars shows a significant difference compared to not cocultured FaDu cells. Black bars showed coculture of FaDu cells with CAFs by the presence of cisplatin. Cisplatin showed significantly different efficacy in inhibiting the formation of the FaDu colonies after coculture with individual patient-derived CAFs. (**c**) Effect of cisplatin. Group comparison (paired test) between coculture with no treatment and coculture in presence of cisplatin showed a significant decrease in the total area of FaDu colonies in the treated group. *p*-values from group comparisons based on the t-test are shown. Asterisks represent statistical significance (* *p* < 0.05; ** *p* < 0.01; *** *p* < 0.001). (**d**) The sensitizing ratio showing the extent of inhibition of colony-forming capacity after cisplatin treatment in cancer cells cocultured with patient-derived CAFs. Values above 0 indicate a higher level of inhibition when exposed to cisplatin. The control corresponded to sensitizing ratio of non-cocultured cells (equal to 0).

To assess if CAF-derived compounds support head and neck cancer cell growth, CAFs were cocultured with FaDu cell line in a transwell system. CAFs derived from tumor tissue of individual HNSCC patients significantly differed in their ability to support or inhibit the colony-forming capability of FaDu cells (Figure 2a,b, Supplementary Table S1). Patient-derived early passage CAFs M.5.1 significantly inhibited the clonogenic survival of FaDu cells. On the other hand, patient-derived early passage CAFs M.6.1, M.7.1, and M.11.1 significantly supported the clonogenic capacity of FaDu cells. These were further named as tumor-supporting and tumor-inhibiting CAFs. 

To test a hypothesis that CAFs may affect the sensitivity of cancer cells to cisplatin, CAF-FaDu coculture was exposed to 5 μM cisplatin treatment. Transwell^®^ cell culture inserts were used, which means the medium was shared between both cell populations (CAFs and cancer cells). Cisplatin was added to the cultivation medium and therefore influenced both cell populations. There was a systematic decrease in colony-forming capability after cisplatin treatment (Figure 2b,c), however, the extent of this decrease differed among CAFs and was expressed as a “sensitizing ratio” (Figure 2d; for calculation see the method section). Of those, CAFs M.5.1 and M6.1 were considered cisplatin-sensitizing.

### 2.4. The Colony-Forming Capability of FaDu Cells after CAF Coculture Is Related to Cancer-Associated Genes

**Figure 3:**
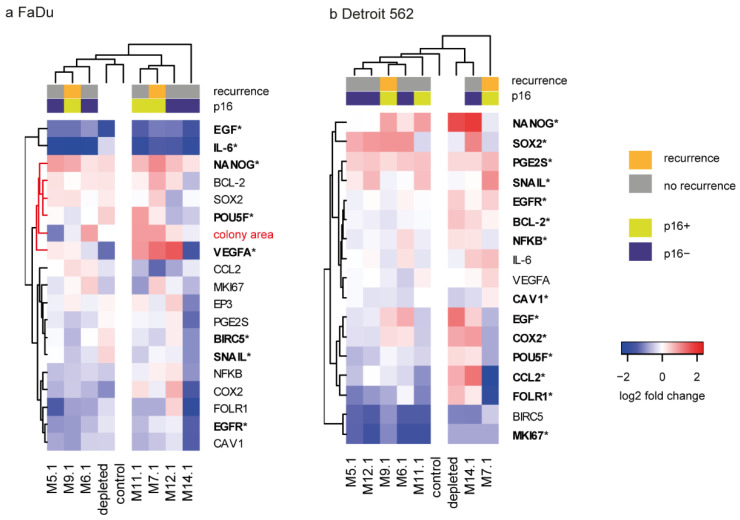
The effect of CAF-derived media (CMCAF) on gene expression in FaDu and Detroit cells. (**a**) Gene expression pattern of FaDu cells relative to non-co-cultured FaDu cells in log2 fold change, together with the log2-transformed colony area. Red cluster branch indicates genes clustered with colony area, see Figure 4d for correlations. (**b**) Gene expression pattern of Detroit 562 cell line cocultured with patient-derived CAFs; the analogue of (**a**). Genes highlighted in bold with an asterisk indicate significant change compared to the 24 h-depleted medium. Columns represent patients; rows represent genes. Gene expression levels are indicated by color: red denotes upregulation; blue denotes downregulation.

Gene expression analysis of FaDu cells was performed to link CAF colony-forming capability with respective signalling in cancer cells. *VEGFA* and *NANOG* expression was increased, and *EGF*, *IL6*, *POU5F*, *BIRC5*, *SNAIL*, and *EGFR* were decreased compared to expression with depleted medium (noted by an asterisk in Figure 3a), suggesting that the effect of CAFs differs from the simple exhaustion of nutrients and/or production of waste metabolites. A similar experiment performed with Detroit cells showed that coculture with CAFs changed expression of another gene set (Figure 3b), suggesting that cancer cell response to CAFs differs among primary tumor cells and metastatic cells.

*NANOG*, a gene associated with pluripotency, was almost uniformly upregulated in FaDu cells when cocultured with media obtained from patient-derived CAFs (compared to control fresh medium) (Figure 3a, Supplementary Tables S2 and S4). Interestingly, cocultured FaDu also showed significantly downregulated expression of *IL6*. Nevertheless, the effect on gene expression in FaDu cells significantly differed between individual CAFs-derived conditioned media. In Detroit cells, uniform upregulation of *PGE2S* and downregulated expression of *BIRC5* and *MKI67* due to the coculture were shown. 

**Figure 4:**
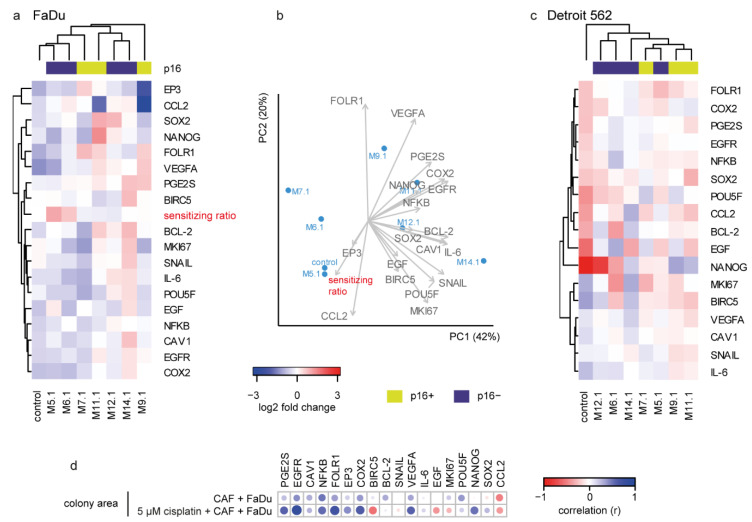
Effect of cisplatin on gene expression and its association with sensitizing ratio. (**a**) Heatmap of gene expression shown as a log2 fold change relative to individual CAF cocultured cisplatin untreated FaDu cells (to remove the among-CAF effect, the gene expression after coculture with each CAF (without cisplatin) was pairwise set as 0). The cisplatin effect on FaDu cell gene expression was CAF-specific and fell into two clusters: a cluster of CAFs M.5.1, M.6.1, and M.7.1 together with the non-cocultured control caused a general expression decrease and increase of cisplatin sensitivity, while M.11.1, M.12.1, and M.14.1 coculture resulted in the opposite; an increase in most of the studied genes and decreased/unchanged sensitizing ratio. (**b**) Principal component analysis of FaDu-cocultured cells. Colony-forming-based “sensitizing ratio”, *EP3*, and *CCL2* were clustered opposite to *VEGFA*, *PGE2S*, *COX2*, *NANOG*, and EGFR. (**c**) Gene expression pattern of Detroit 562 cells cocultured with CAF-derived media; the analogue of (**a**). (**d**) Correlation of colony area with the expression of target genes in FaDu cells cocultured with CAF-derived media, with or without cisplatin. A significant positive correlation is indicated by blue, negative by red. Size of the circle corresponds to significance (bigger circle corresponds to lower *p*-value).

The correlation analysis revealed that the expression of many cancer-associated genes such as *PGE2S*, *EGFR*, *CAV1*, *NFKB*, *FOLR1*, *COX2*, *BCL2*, *VEGFA*, and *POU5F* was closely related and proportional to the size of the area of tumor colonies in coculture experiments and that the area of tumor colonies was in negative correlation with *CCL2* expression (see Figure 4d, Supplementary Table S3). 

Regarding clinicopathological conditions, coculture of FaDu cells with CAFs-conditioned media derived from patients who subsequently underwent recurrence (M.9.1 and M.7.1) significantly increased the *SOX2* expression in FaDu cells compared to coculture with CAFs-conditioned media derived from patients who did not undergo recurrence. No other similar associations with recurrence and/or p16 status were observed in either FaDu or Detroit 562 cells.

### 2.5. Cisplatin Response in FaDu Cells Is CAF-Specific

Similarly to the previous step, gene expression changes in FaDu cells after coculture with medium from cisplatin-influenced CAFs (CMCAF_post-cisplatin) were associated with a colony-forming-based “sensitizing ratio”. To remove the among-CAF effect, the gene expression after coculture with each CAF (without cisplatin) was pairwise set as 0. The cisplatin effect on FaDu cell gene expression was CAF-specific: a cluster of CAFs M.5.1, M.6.1, and M.7.1 together the with non-cocultured control caused a general expression decrease of most genes (Figure 4a) and increase of cisplatin sensitivity, while M.11.1, M.12.1, and M.14.1coculture resulted in the opposite, an increase in most of the studied genes and cisplatin resistance-supporting effect. The expression increase of *VEGFA*, *PGE2S*, *COX2*, *EGFR*, and *NANOG* was characteristic for the increase of resistance, as the sensitizing ratio was in the opposite direction in the principal component analysis (Figure 4b). On the other hand, *CCL2* expression was associated with sensitizing effect.

However, coculture of CAFs with Detroit 562 cells resulted in a different gene expression pattern, in which “sensitizing” and “resistance-supporting” CAFs were not clustered together (Figure 4c). 

Furthermore, individual genes correlated differently with the colony area of FaDu cells cocultured with conditioned media from cisplatin-treated and cisplatin non-treated CAFs. Conditioned media from cisplatin-treated CAFs caused a negative association of FaDu cells colony area with *BIRC5* and *EGF*, and a positive association of colony area with *NANOG* expression, which was not seen in coculture with CAFs-conditioned media without cisplatin (Figure 4d).

### 2.6. Platin Amount Accumulated to and Released from CAFs Relates to Aggressiveness

**Figure 5:**
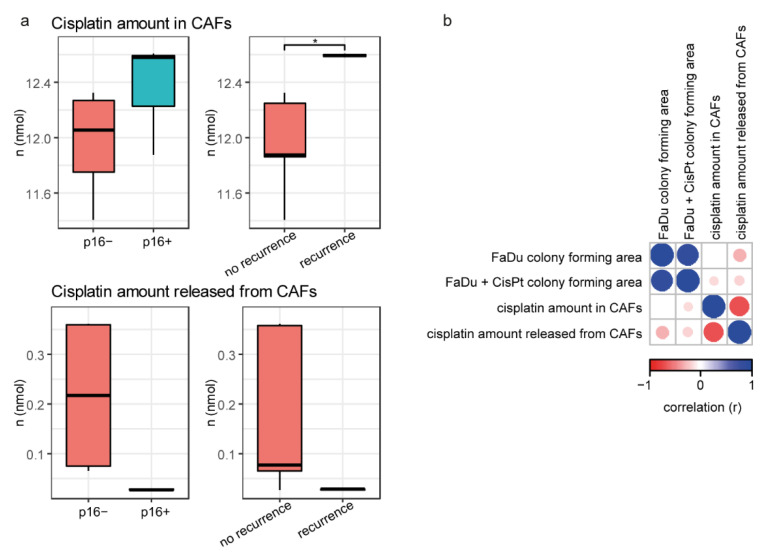
Cisplatin content in media and CAFs. (**a**) Cisplatin amount absorbed from media to CAFs is associated with patient recurrence (higher in CAFs from patients who had a recurrence). Cisplatin amount released from CAFs (CMCAF_post-cisplatin medium) was not associated with p16 status and/or recurrence. (**b**) Correlation heatmap of colony area and cisplatin amount in CAFs and/or cisplatin released from CAFs. Asterisk indicate t-test result with *p* < 0.05.

For the previous gene expression analyses, the CAFs were first incubated with cisplatin (CMCAF+cisplatin), then the medium was changed and conditioned for 24 h (CMCAF_post-cisplatin); see Figure 1c. In both media, the cisplatin content was measured and the amount of cisplatin in cells was approximated as a difference from platin added minus platin measured after 24. The platin amount released from CAFs (Figure 5a) was measured in CMCAF_post-cisplatin medium (cisplatin presented in CMCAF_post-cisplatin medium could only get there by efflux from CAFs). Significantly, higher amounts of cisplatin were found in CAFs derived from patients who subsequently underwent a recurrence (*p* = 0.016). The amount of cisplatin released from CAFs into CMCAF_post-cisplatin medium negatively correlated with the amount of cisplatin absorbed into CAFs in CMCAF+cisplatin medium (Figure 5b) and was also negatively associated with the area of the colonies.

## 3. Discussion

Cancer-associated fibroblasts (CAFs) are one of the most abundant and critical components of the tumor stroma. They provide physical support for tumor cells, but also play a key role in a context-dependent promoting or delaying of cancerogenesis. CAFs can impact angiogenesis, metastasis, immunosurveillance, metabolic reprogramming, treatment resistance, stemness, and remodeling of the extracellular matrix [10]. Some of these effects need the direct contact of CAFs and cancer cells, while some involve paracrine signals [11]. 

Resistance to cisplatin is a major obstacle to improving the chemotherapy outcomes of HNSCC patients. In the present study, we found that cisplatin response in FaDu cells is CAF-specific as different patient-derived CAFs have a different “sensitising ratio” (Figure 2d). Expression increase of *VEGFA*, *PGE2S*, *COX2*, *EGFR*, and *NANOG* in cancer cells was characteristic for the increase of resistance, as the sensitizing ratio was the opposite in the principal component analysis (Figure 4b). On the other hand, *CCL2* expression was associated with sensitizing effect.

We also found that patient-derived CAFs significantly affected the colony-forming capability of cancer cells by changes in expression of cancer-associated genes in tumor cells. The correlation analysis revealed that expression of many cancer-associated genes, such as *PGE2S*, *EGFR*, *CAV1*, *NFKB*, *FOLR1*, *COX2*, *BCL2*, *VEGFA*, and *POU5F*, was closely related and proportional to the size of the area of tumor colonies in coculture experiments. On the other hand, the area of tumor colonies was in negative correlation with *CCL2* expression (see Figure 4d). Cisplatin further enhanced the positive correlation between the area of tumor colonies and gene expression of *PGE2S*, *EGFR*, *FOLR1*, *EP3*, *COX2*, and *VEGFA* in cancer cells, highlighting the importance of these genes in treatment resistance. Furthermore, cisplatin treatment of CAFs caused a negative association of colony area with *BIRC5* and *EGF*, and a positive association of colony area with *NANOG* expression in cancer cells treated by CMCAF_post-cisplatin medium, which was not seen in CMCAF cocultured cells (Figure 4d). Taken together, coculture with CAFs can significantly enhance the expression of genes important for cancer progression. On the other hand, *CCL2* expression seems to have an anti-clonogenic effect in FaDu cells. A similar effect has been observed in animal models, where *CCL2* suppressed tumor growth in a T lymphocyte-independent and/or -dependent manner. CHO cells expressing high levels of either human or murine *CCL2* formed no tumors. Sarcoma cells expressing high levels of *CCL2* appeared later, grew more slowly, and were associated with an intense monocyte/macrophage infiltration [12,13,14]. The negative correlation of colony area with *BIRC5* expression caused by cisplatin may be associated with negative regulation of autophagy by BIRC5. BIRC5 negatively modulates the protein stability of ATG7 and physically binds to the ATG12–ATG5 conjugate, preventing the formation of the ATG12–ATG5-ATG16L1 protein complex [15]. Autophagy can function as a protective cell survival mechanism against environmental and cellular stress, and perhaps causes resistance to anticancer therapies [16], as CAFs were shown to confer cisplatin resistance to tongue cancer via autophagy activation [17]. High amounts of BIRC5 could therefore be undesirable in stressed cancer cells.

Significantly higher amounts of cisplatin were found in CAFs derived from patients who subsequently experienced a recurrence. Consequently, we can speculate that higher ability to accumulate cisplatin leads to higher activation of the tumor-supporting abilities of CAFs after stress exposure. For example, interleukin-11 was significantly up-regulated in the CAFs stimulated by cisplatin. IL-11 facilitated cancer cell chemoresistance via IL-11R/STAT3 signalling [18]. Cisplatin may also activate PAI-1 secretion from CAFs, with paracrine effects promoting esophageal squamous cell carcinoma progression and causing chemoresistance [11]. The amount of cisplatin released from CAFs negatively correlated with the amount of platin absorbed into CAFs (Figure 5b) and was also negatively associated with the area of colonies. Coculture of FaDu cells with CAFs-conditioned media derived from patients who subsequently had a recurrence significantly increased the *SOX2* expression in FaDu cells. Constitutive expression of *SOX2* in HNSCC cells was shown to generate a cancer stem cell-like phenotype [19].

A limitation of our study lies in the small number of studied CAF populations, and the observational and screening-based character of this study. Furthermore, our model cancer cell lines probably cannot fully reflect the whole heterogeneity of HNSCC. Nevertheless, our results showed that CAFs could promote and/or inhibit colony-forming capability and cisplatin resistance in HNSCC cells via paracrine effects, and subsequent changes in the gene expression of cancer-associated genes in cancer cells.

Further studies should be executed to prove our findings and to disclose the exact signaling pathways enabling the cancer-promoting effect of CAFs.

## 4. Materials and Methods

### 4.1. Model Cell Lines

Detroit562 is an epithelial adherent cell line derived from the pleural effusion of metastatic pharyngeal carcinoma which contains the activating PI3K mutation. FaDu is an epithelial adherent cell line derived from hypopharyngeal carcinoma which does not carry the PI3K mutation [20]. FaDu and Detroit562 cell lines were purchased from CLS Cell Lines Service GmbH, Germany. FaDu and Detroit562 cells were cultured in MEM medium with 10% FBS, supplemented with antibiotics (penicillin 100 U/mL and streptomycin 0.1 mg/mL) and 0.4 μg/mL hydrocortisone. Cells were maintained at 37 °C in a humidified incubator with 5% CO_2_ (Sanyo, Japan). The passages of cells ranged from 5 to 10.

### 4.2. Tumor Samples Collection

The study was conducted in accord with the Helsinki Declaration of 1964, and all subsequent revisions thereof. It was approved by the ethical committee of Motol University Hospital, Prague, Czech Republic. All surgical tissue samples were obtained from HNSCC patients after they had signed informed consent. Patients with other malignancies or inflammatory and infectious diseases were excluded from this study. Patients were completely clinically examined. The tumor extent was assessed by physical examination and imaging methods (CT scan and/or MRI). Tumor samples were taken from verified HNSCC under local or general anesthesia; the inclusion criteria were as follows: (a) histologically confirmed squamous cell carcinoma with no distant metastases; (b) no previous oncologic treatment; (c) no other malignancies; (d) no inflammatory or infectious diseases. Tumor grade was evaluated following the World Health Organization (WHO) criteria, and tumor staging was determined using the TNM system (8th edition) of the International Union Against Cancer. All patients underwent surgery followed by radiotherapy or chemoradiotherapy with curative intent. Each tissue specimen underwent pathology quality control. Hematoxylin and eosin-stained sections from each sample were subjected to independent pathology review to confirm squamous cell carcinoma histological consistence. Tumor samples with >60% tumor nuclei and <20% necrosis were considered sufficient for the following analyses. The specimens harvested at surgery were collected in tubes containing culture medium supplemented with antibiotics and antimycotics with the addition of 1% trypsin (described below). The samples were maintained cold, and the primary cell lines were prepared within 24 h. 

### 4.3. Patient-Derived Cell Cultures

Tissue material was placed into the culture medium (MEM) with 1% added trypsin and 1% antibiotic-antimycotic solution (Santa Cruz Biotechnology, Dallas, TX, USA). The following protocol was used to process the primary culture from the sample. The tissue was first rinsed with 50% ethanol (Sigma-Aldrich, St. Louis, MO, USA) followed by phosphate buffer (Invitrogen, Waltham, MA, USA). Necrotic and adipose tissue was then removed from the tissue using a sterile scalpel, and the tumor tissue was cut into 3 mm pieces. Subsequently, the tumor pieces were transferred to a sterile tube with phosphate buffer and centrifuged at 4 °C, 2700 rpm, for 7 min. After removing the supernatant, complete medium (MEM supplemented with 10% FBS, 1% antibiotic and antifungal solution, 10 μg/mL gentamicin sulfate and 10 μg/mL ciprofloxacin) was added to the sample, and the sample was cultured at 37 °C and 5% CO_2_ until the cells reached 50% confluence. After this time, the medium was changed to medium only with the addition of FBS (Biochrom, Cambridge, UK) and Pen/Strep antibiotic solution (Biochrom). 

Since we were primarily interested in TME-derived fibroblasts (CAFs), selection using population overgrowing was performed. To verify the purity of the CAF cell culture, the cells were stained for the mesenchymal surface marker CD90 and analyzed by phase-contrast microscopy and flow cytometry (see Figure 1a,b). Cells were prepared as a single-cell suspension for FACS staining. For CD90, cells were stained with APC-CD90 antibody (Clone #5E10, Biolegend, San Diego, CA, USA) for 30 min at 4 °C. CD90+ and CD90− cells were identified based on isotype gating. The stained cells were acquired for analysis on a FACS Aria II flow cytometer (BD, Franklin Lakes, NJ, USA). Flow cytometry data were analyzed with FlowJo 10.7 (BD).

### 4.4. Colony-Forming Assay

FaDu cell line was cocultured with CAFs derived from the tumor tissue of HNSCC patients by using Transwell^®^ cell culture inserts in a 1:50 ratio. For direct coculture, inserts with a 0.4 µm pore size were used, which ensured that only soluble factors passed from CAFs through the insert to the FaDu cells seeded in the well below. After adhering overnight, the medium was changed for the control medium without cisplatin or medium containing 5 µM cisplatin, and cells were cultivated for 3 weeks to form colonies. The concentration of cisplatin was determined based on the literature [9,21]. Transwell^®^ cell culture inserts were used, which means the medium was shared between both cell populations (CAFs and cancer cells). Cisplatin was added to the cultivation medium and therefore influenced both cell populations.

After cultivation, colonies were fixed with cold methanol and visualized by trypan blue. The colonies were segmented by thresholding of the blue component of image transformed into Lab color space, where a single fixed threshold was used. Finally, the fraction of areas covered by colonies was computed. 

To calculate the extent of inhibition of colony-forming capacity after cisplatin treatment of cocultured cancer cells with patient-derived CAFs, a sensitizing ratio was calculated as follows:

(1) $$sensitizing\;ratio=-\log_{2}\left(\frac{\frac{a_{cis+}^{CAF}}{a_{cis-}^{CAF}}}{\frac{a_{cis+}^{nc}}{a_{cis-}^{nc}}}\right)$$

where $a_{cis+}^{CAF}$ and $a_{cis-}^{CAF}$ correspond to the total colony area of FaDu cells cocultured with CAFs treated or not treated with 5 μM cisplatin. $a_{cis+}^{nc}$ and $a_{cis-}^{nc}$ correspond to non-cocultured FaDu cells with and without cisplatin, respectively. Therefore, sensitizing ratio of non-cocultured cells = 0, values > 0 indicate a sensitizing effect of CAFs on cisplatin effect on FaDu cells, while values < 0 indicate a resistance-supporting effect.

### 4.5. Gene Expression Analysis

Gene expression assays did not use Transwell^®^ cell culture inserts. Conditioned media were used instead of Transwell^®^ cell culture inserts. After the cells (CAFs) reached optimal confluence, their culture media were replaced with fresh media. After 24 h the conditioned media were collected (CMCAF) and replaced with media containing 5 µM cisplatin [9]. After 24 h the media were collected (CMCAF+cisplatin) and replaced with fresh media, which were collected after an additional 24 h (CMCAF_post-cisplatin) (see Figure 1c). Media were frozen at −20 °C until use in the following analyses. 

FaDu cells were seeded into 6-well plates. After achieving 80% confluency, the cells were cocultured with CAF-derived conditioned media CMCAF and CMCAF_post-cisplatin, control medium (fresh medium), or depleted medium (24 h on cancer cells without coculture). After 48 h, cells were harvested and used for RNA isolation. Cancer cells were not in direct contact with cisplatin.

### 4.6. RNA Isolation and Reverse Transcription

TriPure Isolation Reagent (Roche, Basel, Switzerland) was used for RNA isolation. The isolated RNA was used for cDNA synthesis. RNA (1000 ng) was transcribed using a Transcriptor first-strand cDNA synthesis kit (Roche, Switzerland), which was used according to the manufacturer’s instructions. cDNA (20 μL) prepared from total RNA was diluted with RNase-free water to 100 μL, and the amount of 5 μL was directly analyzed.

### 4.7. Quantitative Real-Time Polymerase Chain Reaction

qRT-PCR was performed using TaqMan gene expression assays with the LightCycler^®^480 II System (Roche, Basel, Switzerland), and the amplified cDNA was analyzed by the comparative Ct method using *ACTB* and *PSMB2* [22] as housekeep controls. Primer and probe sets for *ACTB* (assay ID: Hs99999903_m1), *PSMB2* (Hs01009704_m1), *NANOG* (Hs04260366_g1), *SOX2* (Hs01053049_s1), *POU5F* (Hs04260367_gH), *SNAIL* (Hs00195591_m1), *FOLR1* (Hs01124177), *MKI67* (Hs00606991_m1), *NFKB1* (Hs00765730_m1), *BCL2* (Hs00608023_m1), *VEGFA* (Hs00900055_m1), *EGF* (Hs01099999_m1), *EGFR* (Hs01076078_m1), *COX2* (Hs00153133_m1), *CCL2* (Hs00907239_m1), *IL6* (Hs00985639_m1), *CAV1* (Hs00971716_m1), *BIRC5* (Hs00153353_m1), *EP3* (Hs00168755_m1), and *PGE2S* (Hs00228159_m1) were selected from the TaqMan gene expression assays (Life Technologies, USA). qRT-PCR was performed under the following amplification conditions: total volume 20 μL, initial incubation at 50 °C/2 min, followed by denaturation at 95 °C/10 min, then 45 cycles at 95 °C/15 s and at 60 °C/1 min.

### 4.8. Cisplatin Concentration in CAF-Derived Media

The concentration of platinum in media was determined by ICP-MS Agilent 7900 (Agilent Technologies, Santa Clara, CA, USA). The samples were 10 times diluted before analysis with MQ water, and the Pt concentration was measured using the isotope, 195Pt. Possible matrix effect was suppressed using an internal standard (Tl solution with a concentration of 200 μg/L).

As the cells were treated with 5 μM cisplatin, the content of cisplatin in CAFs was approximated as follows:

(2) $$n_{cisplatin}^{CAF}=n_{cisplatin}^{added}-n_{cisplatin}^{24h\;post-treatment\;in\;medium}$$

Cisplatin released from CAFs was measured in CMCAF_post-cisplatin medium (24 h incubation with fresh medium after cisplatin was removed). Cisplatin present in this medium could only have got there by efflux from CAFs.

### 4.9. Statistical Analysis

Principal component analysis with two components was used to show relations in multidimensional data, Pearson correlations were performed to calculate correlation coefficients between colony-forming assay and gene expression, a one-sample *t*-test was used to compare gene expression of cocultures vs. controls. R 4.0.2 [23] was used for analysis, with the following packages: ggplot2, [24], gplots 3.0.4 [25], heatmap.plus 1.3 [26], Corrplot 0.84 [27], ggpubr 0.4.0 [28], mixOmics 6.14 [29]. Unless noted otherwise, *p*-level < 0.05 was considered significant.

## Figures and Tables

**Table 1:** Clinico-pathological characterization of head and neck squamous cell carcinomas (HNSCC) patients based on TNM staging and grading (8th revision).

Patient	Gender	Age at Diagnosis	Tumor Location	pT	pN	cM	G	p16	Stage	Surgery + Adjuvant Radiotherapy	Adjuvant Chemotherapy	Recurrence
M5.1	F	59	larynx	4a	2b	0	3	0	IVA	1	0	0
M6.1	M	81	oropharynx	2	0	0	2	0	I	1	0	0
M7.1	M	60	hypopharynx	2	2b	0	2	1	IVA	1	0	1
M9.1	M	54	oropharynx	2	1	0	2	1	I	1	1	1
M11.1	M	63	oropharynx	2	2	0	3	1	II	1	1	0
M12.1	M	49	larynx	4a	1	0	3	0	IVA	1	0	0
M14.1	M	62	larynx	4a	0	0	2	0	IVA	1	0	0

## Data Availability

Gene expression, flow cytometry and colony-forming data are available as a supplement of this article.

## Supplementary Materials

The following are available online at https://www.mdpi.com/1422-0067/22/4/1912/s1.
