# Supplementary material for: Sensitivity to Cisplatin in Head and Neck Cancer Cells Is Significantly Affected by Patient-Derived Cancer-Associated Fibroblasts
Source: Int J Mol Sci. 2021 Feb 15;22(4):1912. doi: 10.3390/ijms22041912 (PMC7918851; doi:10.3390/ijms22041912)
Supplement: Supplementary file 1 [file ijms-22-01912-s001.pdf]

Supplementary File 1. Sensitivity to cisplatin in head and neck cancer cells is significantly affected by patient-derived cancer-associated fibroblasts.

Supplementary figure 1: Flow cytometric analysis of CD90 status in cancer-associated fibroblast cultures.

Supplementary table 1: Colony area of FaDu cells non-cocultured (ctrl), co-cultured and treated with cisplatin and respective sensitizing ratio.

Supplementary table 2: Gene expression fold changes of cells affected to co-culture media relative to the depleted medium.

Supplementary table 3: Correlation matrix of log2 gene expression with sensitising ratio (colony-forming assay).

Supplementary table 4: log2 fold change values of FaDu and Detroit 562 cells exposed to co-culture and/or cisplatin treatment.

Supplementary figure 1: Flow cytometric analysis of CD90 status in cancer-associated fibroblast cultures. Forward scatter/side scatter (left) and FL2-CD90 histogram (right) with gating based on isotype control and non-stained control of each sample. For all samples, CD90-staining (first line), isotype control (middle line) and negative staining was tested.

CAF culture 1 CD90

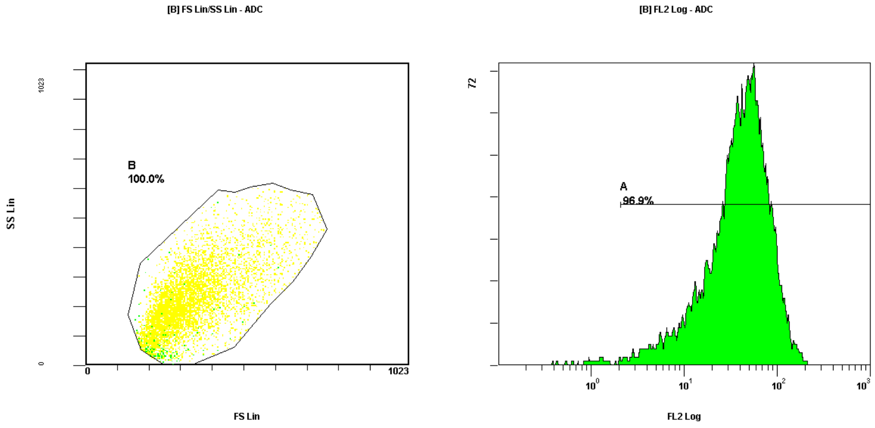

CAF culture 1 isotype control

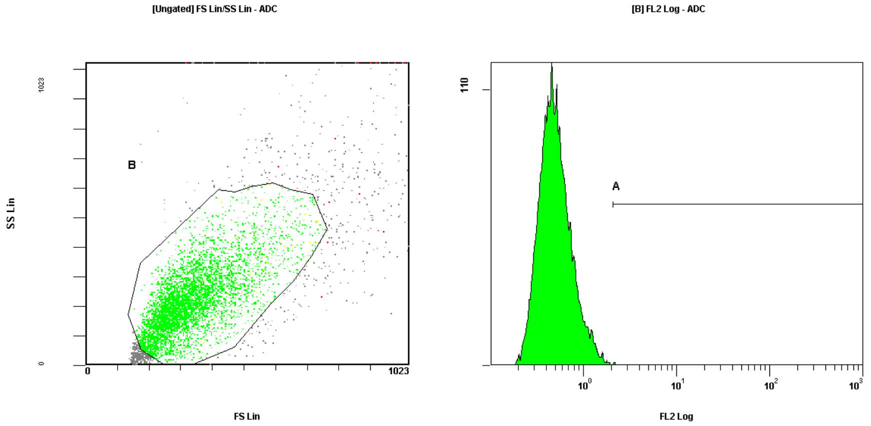

CAF culture 1no staining

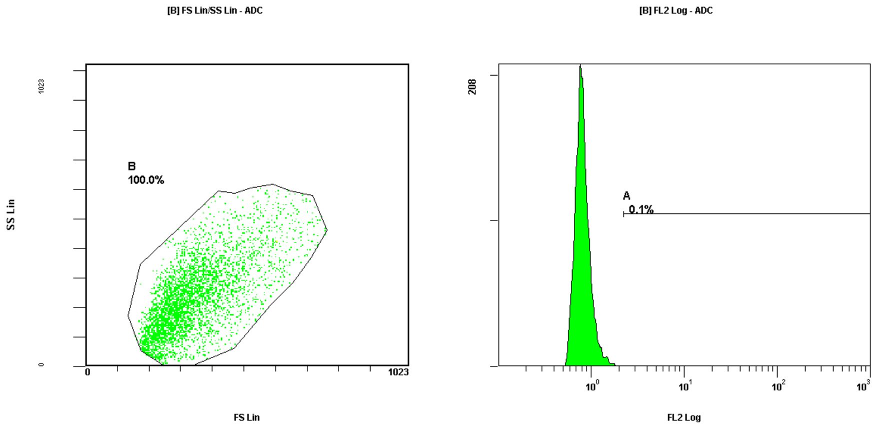

CAF culture 2 CD90

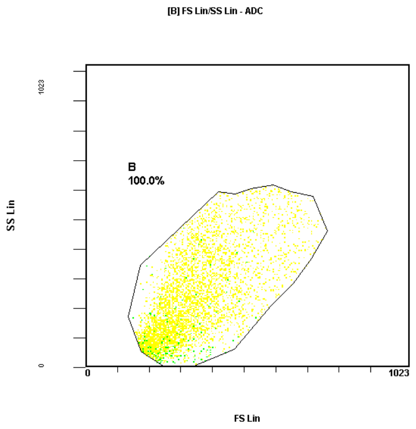

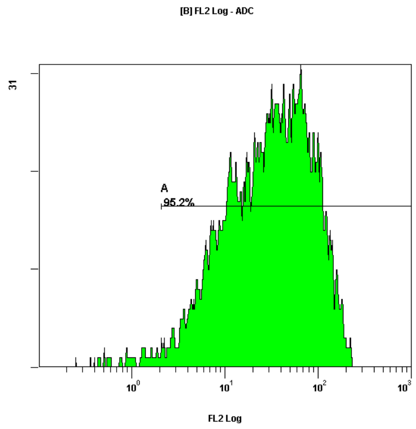

CAF culture 2 isotype control

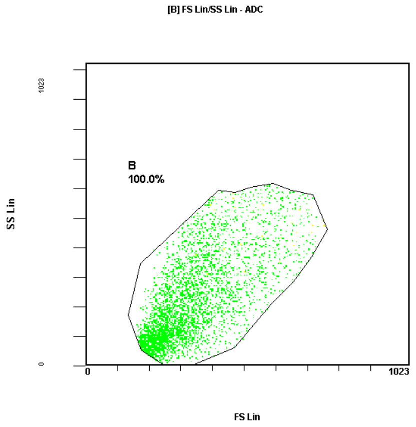

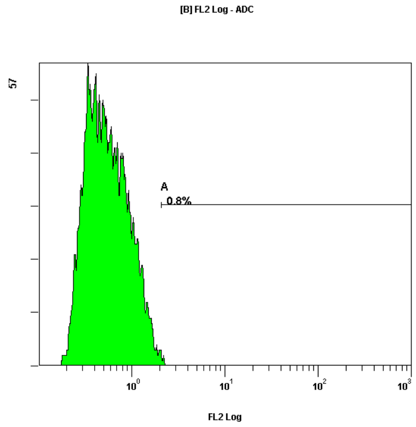

CAF culture 2 no staining

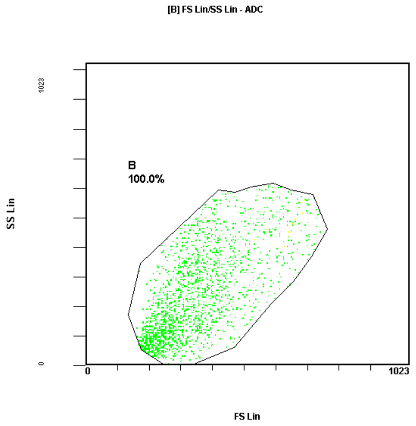

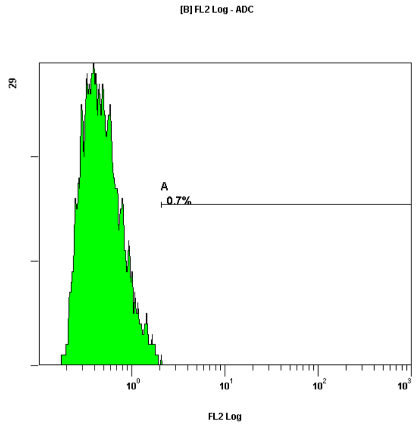

CAF culture 3 CD90 status

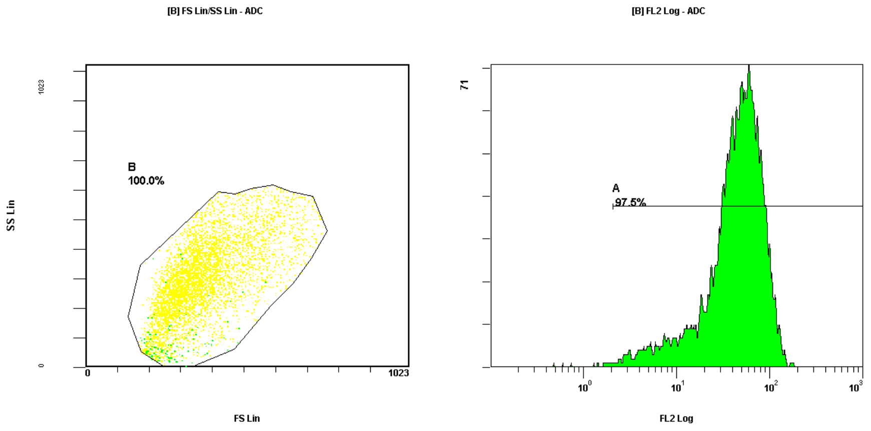

CAF culture 3 isotype control

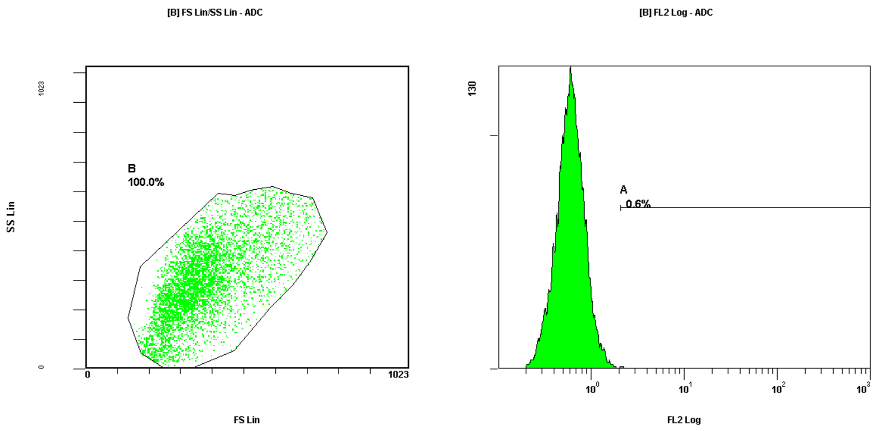

CAF culture 3 no staining

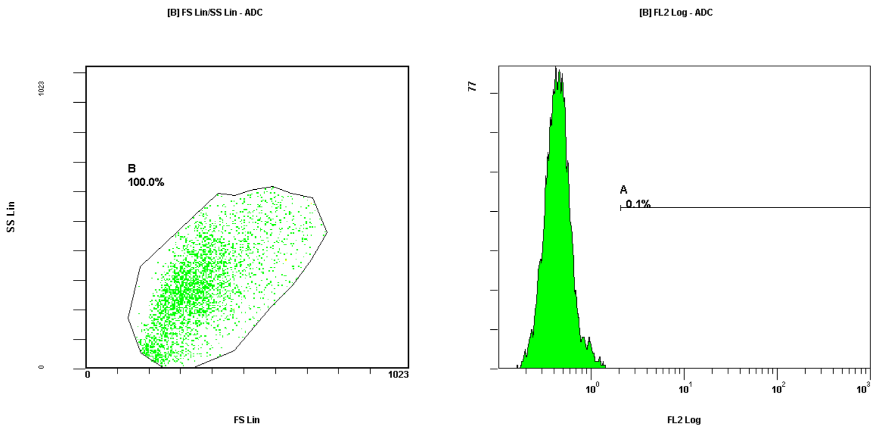

Supplementary table 1: Colony area of FaDu cells non-cocultured (ctrl), co-cultured and treated with cisplatin and respective sensitizing ratio. CAF, cancer-associated fibroblasts, IQR, interquartile range. Calculation of Sensitizing ratio explained in Methods.

patient-derived CAF	FaDu	FaDu + 5uM cisplatin	sensitizing ratio, median, IQR
	well area covered by cells, median, IQR (%)	well area covered by cells, median, IQR (%)	
ctrl	11.03 (10.8 to 12.98)	5.06 (4.29 to 6.75)	0.16 (-0.2 to 0.43)
5.1.	4.81 (3.72 to 6.87)	1.2 (0.92 to 1.48)	1.08 (0.81 to 1.54)
6.1.	21.72 (20.58 to 23.48)	6.44 (5.97 to 6.66)	0.68 (0.63 to 0.79)
7.1.	24.55 (20.57 to 25.87)	13.44 (11.08 to 13.47)	-0.34 (-0.35 to -0.03)
9.1.	11.66 (10.43 to 11.7)	4.68 (3.55 to 6.13)	0.11 (-0.23 to 0.59)
11.1.	21.56 (20.51 to 24.6)	15.65 (10.4 to 16.01)	-0.55 (-0.59 to 0.25)
12.1.	14.44 (13.97 to 15.31)	7.92 (7.54 to 8.93)	-0.21 (-0.37 to -0.14)
14.1.	10.49 (9.7 to 11.81)	4.8 (3.95 to 5.84)	0.07 (-0.19 to 0.39)

Supplementary table 2: Gene expression fold changes of cells affected to co-culture media relative to the depleted medium. IQR, interquartile range.

Detroit 562				Fadu			
	gene	median fold change (IQR)	p-value		gene	median fold change (IQR)	p-value
	VEGFA	1 (0.9 to 1.11)	0.904		VEGFA	2.81 (2.32 to 5.44)	0.008
	MKI67	0.59 (0.41 to 0.9)	0.021		MKI67	1.31 (1.06 to 1.73)	0.103
	EGFR	0.67 (0.6 to 0.71)	0.001		EGFR	0.76 (0.62 to 0.89)	0.032
	SNAIL	1.31 (1.08 to 1.9)	0.043		SNAIL	0.63 (0.54 to 0.7)	0.001
	FOLR1	0.24 (0.2 to 0.4)	0.002		FOLR1	0.71 (0.56 to 0.74)	0.086
	IL-6	1.21 (1 to 1.61)	0.089		IL-6	0.32 (0.28 to 0.39)	0.000
	BCL-2	0.55 (0.54 to 0.63)	0.000		BCL-2	0.98 (0.9 to 1.03)	0.599
	BIRC5	0.78 (0.7 to 1.06)	0.549		BIRC5	0.72 (0.58 to 0.83)	0.011
	CCL2	0.36 (0.25 to 0.46)	0.028		CCL2	1.04 (0.73 to 1.29)	0.691
	CAV1	1.22 (1.16 to 1.47)	0.007		CAV1	0.82 (0.74 to 0.96)	0.083
	EGF	0.26 (0.22 to 0.45)	0.001		EGF	1.58 (1.47 to 1.64)	0.000
	NFKB	0.65 (0.64 to 0.83)	0.010		NFKB	1.09 (0.97 to 1.41)	0.317
	NANOG	0.26 (0.2 to 0.45)	0.005		NANOG	1.21 (1.06 to 1.41)	0.043
	SOX2	2.86 (1.65 to 2.98)	0.021		SOX2	0.85 (0.84 to 1.05)	0.264
	POU5F	0.51 (0.43 to 0.63)	0.003		POU5F	0.72 (0.57 to 0.79)	0.042
	PGE2S	1.16 (1.04 to 1.2)	0.026		PGE2S	0.82 (0.71 to 0.88)	0.061
	COX2	0.36 (0.33 to 0.62)	0.007		COX2	1.46 (1.27 to 2.14)	0.061
					EP3	0.85 (0.83 to 1.04)	0.289

Supplementary table 3: Correlation matrix of log2 gene expression with sensitising ratio (colony-forming assay). Asterisk indicate correlation p-value < 0.05.

FaDu cells + cisplatin, Correlation coefficient

	**VEGFA**	**MKI67**	**EGFR**	**SNAIL**	**FOLR1**	**IL-6**	**BCL-2**	**BIRC5**	**CCL2**	**CAV1**	**EGF**	**NFKB**	**NANOG**	**SOX2**	**POU5F**	**PGE2S**	**COX2**	**EP3**	**colony ratio**
VEGFA	1*	-0.71*	0.61	-0.32	0.89*	-0.16	0.42	-0.7	-0.65	0	-0.59	0.72*	0.66	0.35	-0.14	0.9*	0.82*	0.57	0.59
MKI67	-0.71*	1*	-0.14	0.8*	-0.61	0.65	-0.17	0.82*	0.77*	0.63	0.49	-0.14	-0.42	-0.16	0.34	-0.66	-0.32	-0.18	-0.3
EGFR	0.61	-0.14	1*	0.28	0.77*	0.52	0.14	-0.43	-0.46	0.61	-0.3	0.7	0.58	0.25	0.36	0.77*	0.85*	0.4	0.81*
SNAIL	-0.32	0.8*	0.28	1*	-0.24	0.85*	0.08	0.54	0.4	0.88*	0.4	0.14	0.01	0.13	0.57	-0.18	0.18	0.11	-0.14
FOLR1	0.89*	-0.61	0.77*	-0.24	1*	0.01	0.05	-0.57	-0.6	0.09	-0.42	0.79*	0.44	0.07	-0.22	0.92*	0.77*	0.51	0.72*
IL-6	-0.16	0.65	0.52	0.85*	0.01	1*	-0.16	0.32	0.09	0.93*	0.45	0.21	0	-0.1	0.47	0.02	0.38	0.02	0.18
BCL-2	0.42	-0.17	0.14	0.08	0.05	-0.16	1*	-0.4	-0.16	0.13	-0.64	0.21	0.8*	0.86*	0.5	0.25	0.44	0.32	0.08
BIRC5	-0.7	0.82*	-0.43	0.54	-0.57	0.32	-0.4	1*	0.89*	0.26	0.59	-0.11	-0.75*	-0.43	-0.17	-0.69	-0.57	-0.14	-0.56
CCL2	-0.65	0.77*	-0.46	0.4	-0.6	0.09	-0.16	0.89*	1*	0.16	0.31	-0.1	-0.56	-0.13	-0.05	-0.76*	-0.62	0.01	-0.4
CAV1	0	0.63	0.61	0.88*	0.09	0.93*	0.13	0.26	0.16	1*	0.2	0.41	0.22	0.18	0.57	0.1	0.52	0.24	0.29
EGF	-0.59	0.49	-0.3	0.4	-0.42	0.45	-0.64	0.59	0.31	0.2	1*	-0.45	-0.6	-0.43	-0.05	-0.51	-0.38	-0.07	-0.41
NFKB	0.72*	-0.14	0.7	0.14	0.79*	0.21	0.21	-0.11	-0.1	0.41	-0.45	1*	0.32	0.1	-0.16	0.69	0.7	0.54	0.55
NANOG	0.66	-0.42	0.58	0.01	0.44	0	0.8*	-0.75*	-0.56	0.22	-0.6	0.32	1*	0.86*	0.61	0.61	0.74*	0.47	0.53
SOX2	0.35	-0.16	0.25	0.13	0.07	-0.1	0.86*	-0.43	-0.13	0.18	-0.43	0.1	0.86*	1*	0.69	0.19	0.4	0.57	0.27
POU5F	-0.14	0.34	0.36	0.57	-0.22	0.47	0.5	-0.17	-0.05	0.57	-0.05	-0.16	0.61	0.69	1*	-0.04	0.28	0.1	0.2
PGE2S	0.9*	-0.66	0.77*	-0.18	0.92*	0.02	0.25	-0.69	-0.76*	0.1	-0.51	0.69	0.61	0.19	-0.04	1*	0.85*	0.35	0.58
COX2	0.82*	-0.32	0.85*	0.18	0.77*	0.38	0.44	-0.57	-0.62	0.52	-0.38	0.7	0.74*	0.4	0.28	0.85*	1*	0.47	0.63
EP3	0.57	-0.18	0.4	0.11	0.51	0.02	0.32	-0.14	0.01	0.24	-0.07	0.54	0.47	0.57	0.1	0.35	0.47	1*	0.44
colony ratio	0.59	-0.3	0.81*	-0.14	0.72*	0.18	0.08	-0.56	-0.4	0.29	-0.41	0.55	0.53	0.27	0.2	0.58	0.63	0.44	1*

FaDu cells untreated, Correlation coefficient

	VEGFA	MKI67	EGFR	SNAIL	FOLR1	IL-6	BCL-2	BIRC5	CCL2	CAV1	EGF	NFKB	NANOG	SOX2	POU5F	PGE2S	COX2	EP3	colony ratio
VEGFA	1*	0.3	0.48	0.16	0.5	-0.15	0.68*	0.26	-0.58	0.43	0.32	0.84*	0.58	-0.22	0.07	0.59	0.9*	0.63	0.36
MKI67	0.3	1*	0.34	0.23	0.23	-0.05	0.52	0.11	-0.01	0.4	0.51	0.39	0.18	0.21	-0.33	0.18	0.14	0.13	0.21
EGFR	0.48	0.34	1*	0.76*	0.91*	0.72*	0.64	0.74*	-0.4	0.95*	0.47	0.85*	-0.11	-0.16	0.22	0.87*	0.65	0.68*	0.43
SNAIL	0.16	0.23	0.76*	1*	0.6	0.63	0.67*	0.86*	-0.14	0.76*	0.18	0.44	0.03	0.15	0.34	0.84*	0.25	0.58	-0.02
FOLR1	0.5	0.23	0.91*	0.6	1*	0.58	0.47	0.72*	-0.25	0.89*	0.4	0.82*	-0.18	-0.45	0.03	0.83*	0.76*	0.82*	0.38
IL-6	-0.15	-0.05	0.72*	0.63	0.58	1*	0.1	0.48	-0.15	0.64	0.35	0.34	-0.5	-0.02	0.18	0.47	0.13	0.2	0.07
BCL-2	0.68*	0.52	0.64	0.67*	0.47	0.1	1*	0.53	-0.53	0.57	0.13	0.7*	0.6	0.25	0.34	0.75*	0.51	0.6	0.34
BIRC5	0.26	0.11	0.74*	0.86*	0.72*	0.48	0.53	1*	-0.05	0.83*	0.22	0.51	-0.17	-0.29	0.28	0.88*	0.46	0.71*	0.11
CCL2	-0.58	-0.01	-0.4	-0.14	-0.25	-0.15	-0.53	-0.05	1*	-0.15	0.17	-0.61	-0.46	0.09	-0.17	-0.31	-0.44	-0.1	-0.49
CAV1	0.43	0.4	0.95*	0.76*	0.89*	0.64	0.57	0.83*	-0.15	1*	0.62	0.78*	-0.23	-0.23	0.17	0.87*	0.64	0.71*	0.31
EGF	0.32	0.51	0.47	0.18	0.4	0.35	0.13	0.22	0.17	0.62	1*	0.49	-0.3	-0.06	-0.14	0.3	0.44	0.22	0.01
NFKB	0.84*	0.39	0.85*	0.44	0.82*	0.34	0.7*	0.51	-0.61	0.78*	0.49	1*	0.19	-0.29	0.1	0.78*	0.9*	0.68*	0.51
NANOG	0.58	0.18	-0.11	0.03	-0.18	-0.5	0.6	-0.17	-0.46	-0.23	-0.3	0.19	1*	0.39	0.12	0.13	0.21	0.19	-0.06
SOX2	-0.22	0.21	-0.16	0.15	-0.45	-0.02	0.25	-0.29	0.09	-0.23	-0.06	-0.29	0.39	1*	0.45	-0.15	-0.46	-0.26	-0.11
POU5F	0.07	-0.33	0.22	0.34	0.03	0.18	0.34	0.28	-0.17	0.17	-0.14	0.1	0.12	0.45	1*	0.38	0.09	0.24	0.38
PGE2S	0.59	0.18	0.87*	0.84*	0.83*	0.47	0.75*	0.88*	-0.31	0.87*	0.3	0.78*	0.13	-0.15	0.38	1*	0.72*	0.88*	0.25
COX2	0.9*	0.14	0.65	0.25	0.76*	0.13	0.51	0.46	-0.44	0.64	0.44	0.9*	0.21	-0.46	0.09	0.72*	1*	0.78*	0.39
EP3	0.63	0.13	0.68*	0.58	0.82*	0.2	0.6	0.71*	-0.1	0.71*	0.22	0.68*	0.19	-0.26	0.24	0.88*	0.78*	1*	0.2
colony ratio	0.36	0.21	0.43	-0.02	0.38	0.07	0.34	0.11	-0.49	0.31	0.01	0.51	-0.06	-0.11	0.38	0.25	0.39	0.2	1*

Supplementary table 4: log2 fold change values of FaDu and Detroit 562 cells exposed to co-culture and/or cisplatin treatment. All log2 fold changes are calculated relative to untreated control of particular cell line (which log2 fold change is equal to 0). CAF, cancer-associated fibroblast.

**FaDu cells + Cisplatin, log2 fold change**																		
affected_by patient-derived CAFs	VEGFA	MKI67	EGFR	SNAIL	FOLR1	IL-6	BCL-2	BIRC5	CCL2	CAV1	EGF	NFKB	NANOG	SOX2	POU5F	PGE2S	COX2	EP3
no coculture+cisplatin	-1.35	0.09	-0.53	0.09	-1.01	-0.41	-0.48	0.02	-0.47	-0.51	-0.70	-0.55	-0.31	-0.45	-0.16	-0.17	-0.75	-1.00
M11.1+cisplatin	1.35	-0.74	-0.38	-0.38	-0.22	-2.03	0.76	-0.74	-2.43	-0.72	-1.73	-0.48	2.06	1.00	0.34	0.20	0.26	0.25
M12.1+cisplatin	1.71	-0.23	-0.38	-0.09	0.23	-1.15	0.53	-0.18	-0.67	-0.46	-1.70	0.38	0.84	0.15	-0.41	0.20	0.48	0.16
M14.1+cisplatin	-1.65	0.08	-1.06	-0.31	-2.48	-1.56	0.24	-0.15	-0.04	-0.69	-1.31	-0.97	0.16	0.02	-0.05	-0.56	-1.23	-1.75
M5.1+cisplatin	-0.91	-0.32	-1.42	-0.34	-2.17	-2.61	0.27	-0.01	-0.06	-0.96	-0.98	-0.92	-0.09	0.08	-0.51	-0.40	-1.42	-0.62
M6.1+cisplatin	-0.49	-0.40	-0.93	-0.63	-0.98	-3.13	0.09	-0.15	0.43	-1.01	-1.80	-0.37	0.02	-0.01	-0.57	-0.22	-1.47	-0.75
M7.1+cisplatin	1.08	-0.76	-0.78	-0.81	0.12	-2.45	-0.23	-0.39	-1.36	-0.94	-1.15	-0.39	0.14	-0.18	-0.84	-0.04	-0.59	0.37
M9.1+cisplatin	0.81	-0.93	-0.79	-0.82	-0.24	-2.59	-0.01	-0.59	-2.91	-1.04	-1.66	-0.56	0.29	-0.50	-0.73	0.20	-0.46	-2.12
**FaDu cells, untreated, log2 fold change**																		
affected_by patient-derived CAFs	VEGFA	MKI67	EGFR	SNAIL	FOLR1	IL-6	BCL-2	BIRC5	CCL2	CAV1	EGF	NFKB	NANOG	SOX2	POU5F	PGE2S	COX2	EP3
depleted medium	-1.29	-0.51	-0.23	0.40	-0.31	-0.36	0.25	0.26	-0.26	-0.41	-1.89	-0.48	0.34	0.20	0.46	0.07	-1.02	0.11
M11.1	0.95	-0.91	-0.62	-0.47	-0.74	-1.98	0.14	-0.21	-0.63	-0.70	-1.45	-0.15	0.62	-0.04	0.90	-0.09	0.30	0.19
M12.1	1.56	-0.13	-0.15	-0.15	0.41	-1.53	0.30	0.18	-0.81	-0.25	-1.20	0.25	0.57	-0.75	-0.65	0.16	0.73	0.46
M14.1	-1.64	-0.64	-1.43	-0.99	-2.05	-2.00	-0.61	-0.99	-0.21	-1.45	-1.71	-0.97	0.27	-0.06	-0.48	-1.33	-1.59	-1.12
M5.1	0.20	-0.25	-0.95	-0.02	-1.53	-2.20	0.28	-0.05	0.00	-0.79	-1.23	-0.57	0.89	0.25	0.05	-0.21	-0.70	-0.13
M6.1	-0.31	0.45	-0.62	-0.27	-0.81	-2.26	0.22	0.02	0.20	-0.43	-0.98	-0.37	0.26	-0.05	-0.02	-0.32	-0.66	-0.13
M7.1	1.34	0.47	-0.22	-0.09	-0.74	-1.49	0.77	-0.41	-1.35	-0.52	-1.15	0.15	1.08	0.46	0.18	-0.13	-0.16	-0.20
M9.1	0.13	0.07	-0.89	-0.54	-0.86	-2.37	0.04	-0.65	0.32	-0.90	-1.23	-0.49	0.79	0.27	-0.26	-0.52	-0.47	0.15
**Detroit 562 cells, untreated, log2 fold change**																		
affected_by patient-derived CAFs	VEGFA	MKI67	EGFR	SNAIL	FOLR1	IL-6	BCL-2	BIRC5	CCL2	CAV1	EGF	NFKB	NANOG	SOX2	POU5F	PGE2S	COX2	EP3
depleted medium	0.00	-1.09	0.63	-0.10	0.82	-0.16	0.71	-1.49	1.02	-0.61	1.71	0.38	2.31	-0.25	0.45	0.48	1.09	NA
M11.1	0.21	-2.38	-0.05	0.77	-1.47	-0.32	-0.07	-2.00	-1.33	-0.01	-0.65	-0.23	1.18	-0.47	-0.52	0.76	-0.61	NA
M12.1	0.00	-2.38	0.04	0.87	-1.26	0.11	-0.19	-1.99	0.02	-0.35	-0.26	-0.11	-0.01	1.26	-0.68	0.71	-0.39	0.35
M14.1	0.10	-1.09	0.12	0.05	0.27	0.66	0.46	-1.51	1.74	-0.43	0.57	0.32	2.58	1.50	0.36	0.47	1.22	NA
M5.1	-0.07	-1.85	-0.18	0.29	-1.56	-0.02	-0.14	-1.84	-0.76	-0.09	-0.34	-0.29	0.02	1.04	-0.91	0.59	-0.39	-1.27
M6.1	-0.59	-2.37	0.15	-0.04	-0.11	0.39	-0.14	-2.00	-0.47	-0.49	1.03	0.47	0.34	1.32	-0.32	0.68	0.36	2.78
M7.1	0.51	-1.08	0.57	1.40	-2.77	0.81	0.14	-0.63	-3.19	0.23	-0.91	-0.29	-0.28	-0.53	-1.06	0.88	-1.06	NA
M9.1	-0.22	-1.42	-0.20	-0.16	-1.01	-0.34	-0.34	-1.30	-0.27	-0.32	0.57	-0.23	1.13	1.33	-0.12	0.45	0.42	NA
**Detroit 562 cells + Cisplatin, log2 fold change**																		
affected_by patient-derived CAFs	VEGFA	MKI67	EGFR	SNAIL	FOLR1	IL-6	BCL-2	BIRC5	CCL2	CAV1	EGF	NFKB	NANOG	SOX2	POU5F	PGE2S	COX2	EP3
no coculture+cisplatin	-0.36	-1.08	0.62	-0.20	0.80	-0.86	1.15	-1.27	1.31	-0.47	1.99	0.52	3.49	0.78	1.50	0.82	0.79	NA
M11.1+cisplatin	0.67	-2.39	-0.07	1.12	-1.28	-0.11	0.09	-1.24	-0.54	0.04	0.13	0.03	0.28	0.59	-0.49	1.22	-0.94	NA
M12.1+cisplatin	0.08	-2.48	-0.09	0.70	-1.22	-0.27	-0.71	-2.61	-0.02	-0.45	-0.52	0.12	2.72	1.87	0.05	0.74	0.19	0.84
M14.1+cisplatin	-0.53	-2.21	0.05	0.26	0.07	0.66	-0.25	-2.32	0.70	-0.82	1.74	0.23	1.93	1.82	0.51	0.38	1.00	NA
M5.1+cisplatin	-0.22	-1.55	-0.06	0.15	-0.66	-0.30	-0.13	-1.52	-0.99	0.09	0.34	-0.02	0.28	1.26	-0.44	0.61	0.06	NA
M6.1+cisplatin	-0.76	-0.89	-0.21	-0.02	-0.14	-0.03	1.22	-1.19	-0.07	-0.34	-0.07	-0.34	1.60	1.42	0.24	0.33	0.40	0.10
M7.1+cisplatin	0.74	-0.04	0.36	1.06	-2.36	0.61	0.07	-0.32	-2.59	0.25	-1.40	-0.13	0.02	-0.69	-1.76	0.76	-0.77	-1.21
M9.1+cisplatin	-0.02	-0.89	-0.22	0.27	-0.50	-0.01	-0.10	-0.77	0.15	-0.12	0.16	-0.05	-0.07	1.33	-0.63	0.50	0.23	NA
